# Prospective application of clinician-performed lung ultrasonography during the 2009 H1N1 influenza A pandemic: distinguishing viral from bacterial pneumonia

**DOI:** 10.1186/2036-7902-4-16

**Published:** 2012-07-10

**Authors:** James W Tsung, David O Kessler, Vaishali P Shah

**Affiliations:** 1 Division of Pediatric Emergency Medicine, Departments of Pediatrics and Emergency Medicine, Bellevue Hospital Center/NYU School of Medicine, New York, 10016, USA; 2 Departments of Emergency Medicine and Pediatrics, Mount Sinai School of Medicine, 1 Gustave Levy Place, New York, NY, 10029, USA; 3 Department of Emergency Medicine, Childrens Hospital at Montefiore, Albert Einstein College of Medicine, Bronx, NY, 10467, USA; 4 Department of Pediatrics, Columbia University College of Physicians and Surgeons, New York, NY, 10032, USA

**Keywords:** Ultrasound, H1N1 virus, Pneumonia, Emergency medicine, Point-of-care, Pandemic, Pediatric

## Abstract

**Background:**

Emergency department visits quadrupled with the initial onset and surge during the 2009 H1N1 influenza pandemic in New York City from April to June 2009. This time period was unique in that >90% of the circulating virus was surveyed to be the novel 2009 H1N1 influenza A according to the New York City Department of Health. We describe our experience using lung ultrasound in a case series of patients with respiratory symptoms requiring chest X-ray during the initial onset and surge of the 2009 H1N1 influenza pandemic.

**Methods:**

We describe a case series of patients from a prospective observational cohort study of lung ultrasound, enrolling patients requiring chest X-ray for suspected pneumonia that coincided with the onset and surge of the 2009 H1N1 influenza pandemic.

**Results:**

Twenty pandemic 2009 H1N1 influenza patients requiring chest X-ray were enrolled during this time period. Median age was 6.7 years. Lung ultrasound via modified Bedside Lung Ultrasound in Emergency protocol assisted in the identification of viral pneumonia (*n* = 15; 75%), viral pneumonia with superimposed bacterial pneumonia (*n* = 7; 35%), isolated bacterial pneumonia only (*n* = 1; 5%), and no findings of viral or bacterial pneumonia (*n* = 4; 20%) in this cohort of patients. Based on 54 observations, interobserver agreement for distinguishing viral from bacterial pneumonia using lung ultrasound was *ĸ* = 0.82 (0.63 to 0.99).

**Conclusions:**

Lung ultrasound may be used to distinguish viral from bacterial pneumonia. Lung ultrasound may be useful during epidemics or pandemics of acute respiratory illnesses for rapid point-of-care triage and management of patients.

## Background

**Figure 1:**
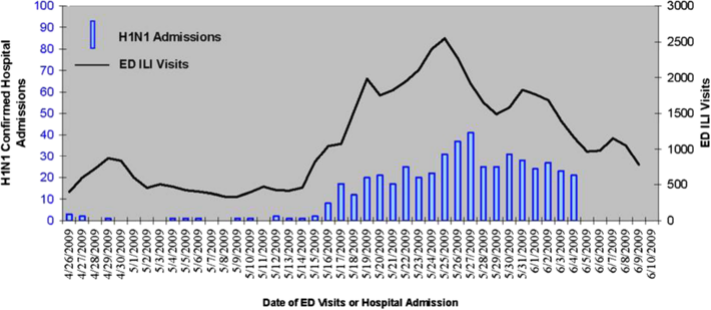
**Laboratory-confirmed H1N1 hospital admissions and emergency department visits for influenza-like illnesses in NYC.** 26 April to 10 June 2009. ED visits quadrupled at peak surge. Adapted from [1].

**Figure 2:**
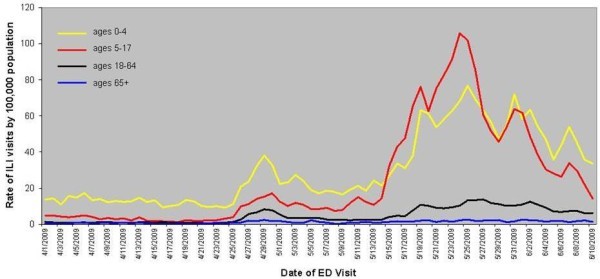
**Rate of influenza-like illness syndrome visits to NYC emergency departments by age group.** Based on chief complaint. 01 April to 01 June 2009. Adapted from [1].

Emergency department visits quadrupled with the initial onset and surge during the 2009 H1N1 influenza pandemic in New York City (NYC) from April to June 2009 (Figures 1 and 2) [1,2]. This time period was unique in that >90% of the circulating virus was surveyed to be the novel 2009 H1N1 influenza A according to the New York City Department of Health. Five-hundred sixty-seven patients requiring hospitalization were confirmed with the 2009 H1N1 influenza A in NYC [1]. In NYC, there were 16 deaths, 46% of admitted patients were <18 years old and 20% were <5 years old [2]. Eighty percent of confirmed cases had a known underlying risk condition, most commonly asthma (40% of confirmed cases) [1].

This fourfold increase in patient volume presented logistical challenges for emergency departments [1]. In response to mass casualty incident-type conditions and overcrowding, emergency departments in New York City added staffing and created alternate sites of care to accommodate the increased patient volume. Increased demand for chest radiography for those patients with more severe disease led to increased delays and length of stay for those patients with suspected, but non-severe pneumonia.

**Figure 3:**
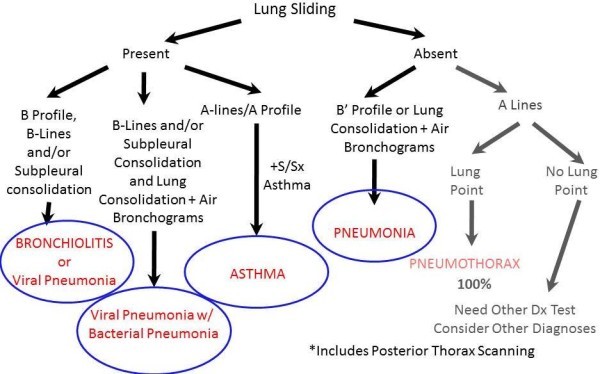
**Bedside Lung Ultrasound in Emergency protocol - modified for ED.** Includes posterior thorax scanning.

Clinicians are challenged by the diagnostic dilemma that influenza cannot reliably be distinguished from other acute respiratory illnesses on the basis of clinical presentation alone [3]. Rapid viral antigen testing for diagnosis, which under ideal situations can yield results within 30 min, is not practical nor cost-effective in pandemic conditions [3]. Point-of-care ultrasound has been demonstrated to identify, in real-time, various pathologies of the lung, such as pneumonia, viral pneumonia, and acute respiratory distress syndrome (ARDS) [4-10] An algorithm for differentiating between various respiratory pathologies has been described (Figure 3) [4], and evidence-based recommendations regarding the use of point-of-care lung ultrasound have recently been published [11]. The use of lung ultrasound during the 2009 H1N1 influenza pandemic in adults has also been recently described [12]. We describe a prospective case series of children in whom clinician-performed lung ultrasonography was used to differentiate between different respiratory pathologies and assessed interobserver agreement of these ultrasound findings during the initial onset and surge of the 2009 H1N1 pandemic (April to June 2009).

## Methods

### Study design and setting

We describe a subcohort of patients who required chest X-ray for suspected pneumonia and were enrolled into a prospective study of lung ultrasound for diagnosing pneumonia that coincided with the onset and surge of the 2009 H1N1 influenza pandemic from April to June 2009 [1,2,13]. We also describe the application of a modified Bedside Lung Ultrasound in Emergency (BLUE) protocol [4] with posterior thorax scanning (Figure 3) during the onset and surge of pandemic patients in an urban emergency department.

This study was approved by our institutional review board. The study population consisted of a convenience sample of patients who met predetermined inclusion criteria and in whom informed consent had been obtained and documented from the patient or guardian for enrollment into the study.

### Selection of participants

Inclusion criteria consisted of patients < 21 years of age presenting to the emergency department with clinical suspicion of pneumonia requiring chest X-ray for evaluationWe excluded those patients who presented the following: (1) arrival in the emergency department with a chest X-ray, (2) a confirmed diagnosis of pneumonia by diagnostic imaging, or (3) hemodynamic instability.

### Methods of measurement and outcome measures

**Figure 4:**
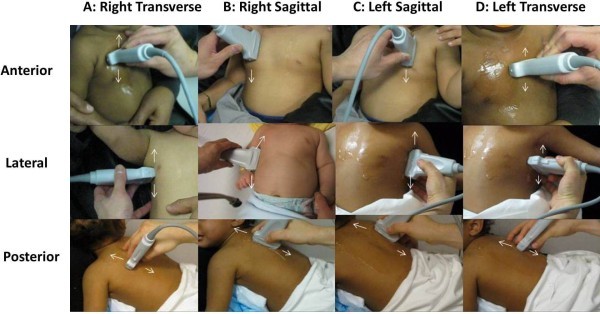
**Six-zone lung scanning protocol.** Top Row: Anterior Midclavicular Line; Middle Row: Lateral Midaxillary Line; Bottom Row: Posterior Paraspinal Line. Probes in transverse (columns in **A** and **D**) and parasagittal planes (columns **B** and **C**) in anterior and posterior lung fields, and in transverse and coronal planes (middle row) in lateral lung fields.

Enrolled patients had a screening history and physical examination performed at the time of triage to determine eligibility into the study. After informed consent was obtained, enrolled patients had clinical exam findings documented on a standardized form and underwent point-of-care lung ultrasound examination. An ultrasound machine with a linear array transducer at 7.5 to 10 MHz (Sonosite Micromaxx, Bothell, WA, USA) was used to image the lungs in perpendicular planes (transverse, parasagittal, and coronal) in the midclavicular line anteriorly and posteriorly on the chest and the midaxillary line from the axillae to diaphragm (Figure 4).

**Figure 5:**
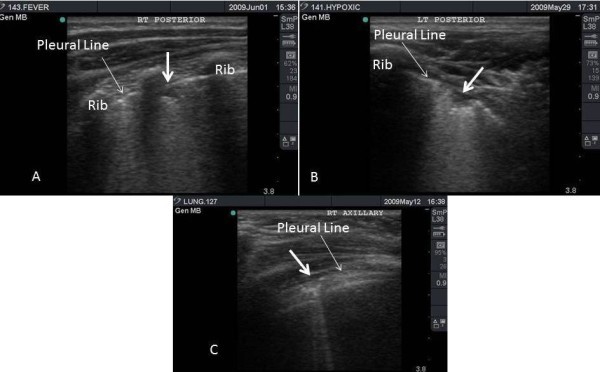
**Small subpleural consolidations (arrows) with trailing comet tail artifacts consistent with viral pneumonia lung ultrasound pattern.** (**A**, **B**, and **C**) are images of small subpleural lung consolidations in three different patients with suspected H1N1.

**Figure 6:**
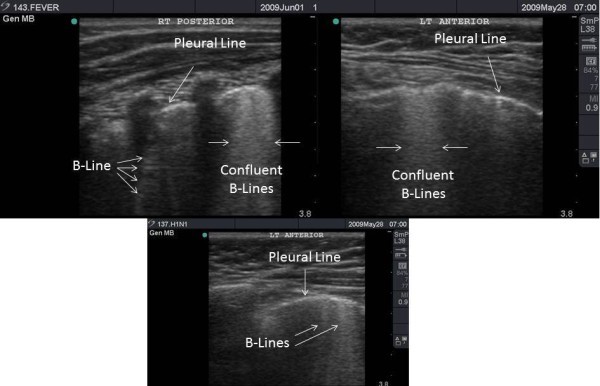
B-lines and confluent B-lines consistent with viral pneumonia lung ultrasound pattern.

**Figure 7:**
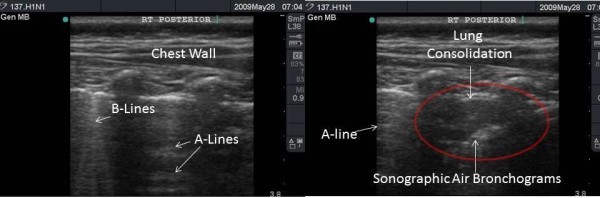
**Viral (B-lines) and bacterial pneumonia (lung consolidation with sonographic air bronchogram) pattern.** A-lines are horizontal lines that represent the normal aerated lung.

Using a six-zone lung ultrasound scanning protocol similar to that described by Copetti et al. [7], we defined and classified patients as positive or negative for viral pneumonia based on the presence of small subpleural consolidations usually <0.5 cm (Figure 5 and Additional file 1) and/or individual B-lines or confluent B-lines (echogenic vertical lines arising from the pleural line to the bottom of the ultrasound screen; Figure 6 and Additional file 2) [7]. These ultrasound findings are similar to those described in interstitial syndrome which is defined as three or more B-lines in a given lung region [10,14,15]. A-lines (horizontal, reverberation artifacts of the pleural line; Figure 7 left) which indicate areas of the normal lung were also noted when present [10,14]. Patients were classified as positive or negative for bacterial pneumonia based on the presence or absence of lung consolidation with air bronchograms [6,7,16] seen on ultrasound (Figures 7 right, 8, and Additional file 3). A clinical course with follow-up after 2 weeks (via electronic medical record and telephone interview) was used to determine disposition and outcomes of enrolled patients. Clinicians performing and interpreting ultrasound were blinded to chest X-ray results, and when performed per hospital protocol for possible admission, viral antigen testing results. Bacterial pneumonia on chest X-ray (posterior-anterior and lateral views) was classified based on the attending pediatric radiologist reading for ‘consolidation, ‘infiltrate, or ‘pneumonia. For analysis purposes only, viral pneumonia on chest X-ray was defined as ‘peri-bronchial cuffing, ‘peri-bronchial thickening, or ‘increased interstitial markings identified by the pediatric radiologist. Pediatric radiologists were blinded to the lung ultrasound results.

**Figure 8:**
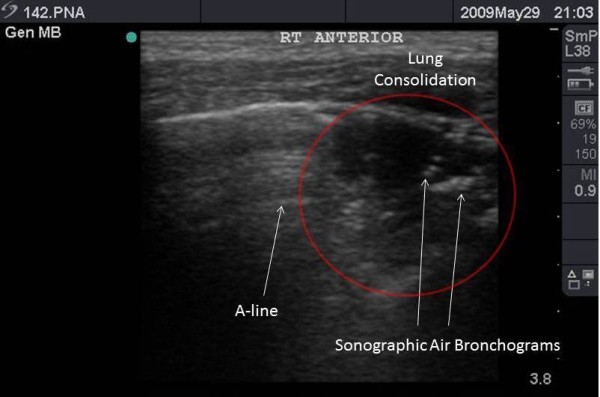
Lung consolidation with sonographic air bronchograms consistent with bacterial pneumonia.

Ultrasound images and videos were reviewed between two blinded investigator sonologists (enrolling sonologist and reviewing sonologist) to determine interobserver agreement by unweighted Cohens Kappa for viral pneumonia (small subpleural consolidation and/or B-lines), normal lung ultrasound pattern (A-lines), and bacterial pneumonia (lung consolidation with sonographic air bronchograms).

## Results

### Characteristics of study subjects

Patient demographic and study characteristics are presented in Table 1. Twenty pandemic 2009 H1N1 influenza patients requiring chest X-ray (CXR) were enrolled during this time period.

**Table 1:** Clinical data

**N**	**20**
Average age	6.7 years (IQR, 3.6 to 10.7)
Gender	65% female
Median US exam time (IQR)	6 min (IQR, 4 to 8)
History of fever	95% (19/20)
History of cough	95% (19/20)
Median time to CXR from request prior to pandemic (*N* = 20)	29 min (IQR, 18 to 43)
Median time to CXR from request during pandemic surge (*N* = 20)	98 min (IQR, 79 to 125)

IQR, interquartile range.

## Main results

Distribution of diagnoses based on lung ultrasound findings, chest X-ray findings, and clinical outcomes using a modified BLUE protocol [4] is presented in Table 2. Interobserver agreement for ultrasound findings of lung consolidation with air bronchograms, B-lines or small subpleural consolidations, and A-lines by Cohen’s Kappa was 0.82 (95% confidence interval (CI), 0.63 to 0.99) (Table 3).

**Table 2:** Main results

**Findings ( *N* ** **= 20)**	**US - *n* ; % [95% CI]**	**CXR - *n* ; % [95% CI]**	**Disposition**^**a**^
Viral pneumonia	15; 75 [53 to 89]	8; 40 [22 to 61]	Oseltamivir
Bacterial pneumonia only	1; 5 [0 to 25]	5; 25 [11 to 47]	Antibiotics and oseltamivir
Viral and bacterial pneumonia	7; 35 [18 to 59]	3; 15 [3 to 38]	Antibiotics and oseltamivir
No findings	4; 20 [7 to 42]	7; 35 [18 to 59]	Discharge and observation

US, ultrasound; CXR, chest X-ray; ^a^Disposition based on US findings.

**Table 3:** Cohen’s Kappa for distinguishing viral from bacterial pneumonia on lung ultrasound between two blinded sonologists

	**Viral Pneumonia**	**Normal**	**Bacterial Pneumonia**	
Viral Pneumonia	13	1	0	14
Normal	1	4	0	5
Bacterial Pneumonia	0	1	7	8
	14	6	7	27
Cohen’s Kappa = 0.82		95% CI (0.63 to 0.99)		

Ultrasound findings of lung consolidation with sonographic air bronchograms [6,7,16] correlated 100% with chest X-ray findings of bacterial pneumonia (reported as consolidation or infiltrate) in eight patients. All of these patients were confirmed to have pneumonia based on the clinical course at 2-week follow-up. This represented a doubling (40% vs. 20%) in the prevalence rate of bacterial pneumonia in our study during the H1N1influenza A onset and surge time period compared to the time period prior to the onset of H1N1 influenza A. The prevalence of viral lung ultrasound findings increased from approximately 50% for the overall study [13] to 75% during the surge of H1N1 influenza. Chest X-ray findings for viral pneumonia (most commonly described as peri-bronchial thickening or peri-bronchial cuffing) were present in 8 of 15 (53%) patients identified as having viral pneumonia on ultrasound. Seven of these 15 patients with viral pneumonia based on ultrasound had superimposed bacterial pneumonia also identified by ultrasound (Figure 7 and Additional file 4). All four patients in our series that required hospitalization had viral and bacterial pneumonia based on ultrasound.

All patients in our series were recovering or recovered from their influenza illness on follow-up after 2 weeks. All admitted patients were subsequently confirmed with the 2009 H1N1 influenza A by the New York City Department of Health. Per hospital protocol for possible hospital admission, four of nine patients tested positive for influenza A by viral antigen testing, despite the New York City Department of Health reporting >90% of the circulating virus during this pandemic time period was the novel influenza A H1N1 [1]. One infant in the cohort was co-infected with respiratory syncytial virus based on viral antigen testing. Three patients, all <5 years of age requiring hospital admission had evidence of both bacterial and viral pneumonia on ultrasound. The only patient requiring ICU admission, a 20-year-old female, was intubated after deteriorating during her ED stay with persistent hypotension and septic shock from a left lower lobe bacterial pneumonia. This patient initially presented with an influenza-like illness and acute abdominal pain.

## Discussion

To our knowledge, this is the first prospective series describing the use of lung ultrasound in children as a potential real-time diagnostic triage tool during a mass casualty-type incident due to an acute respiratory illness pandemic surge [17,18]. Testa et al. have reported on similar lung ultrasound findings in adults during the 2009 H1N1 influenza A pandemic [12]. Single case reports of clinician-performed lung ultrasound to monitor the progression of H1N1 influenza-associated ARDS [19] and point-of-care echocardiography to diagnose H1N1 influenza myocarditis [20] have been described. Retrospective reports of the role of ultrasound in mass casualty incidents during disasters such as earthquakes have also been described [21,22]. Lichtenstein et al. described an algorithm using lung ultrasonography to distinguish between various respiratory pathologies of the lung [4]. We modified Lichtenstein’s BLUE protocol [4] to recognize basic lung ultrasound patterns to distinguish between the normal unaffected lung, viral pneumonia pattern, and bacterial pneumonia (Figure 3). Scanning the posterior thorax was added to increase the sensitivity of the protocol [23]. Point-of-care lung ultrasound was able to identify, in real-time, four groups of pandemic patients: viral pneumonia only (subpleural consolidations and/or B-lines or confluent B-lines), bacterial pneumonia only (lung consolidation with sonographic air bronchograms), both viral and bacterial pneumonia (Figure 7), and normal lungs (A-lines only). Our calculated Kappa was 0.82, which means that the interobserver agreement in distinguishing between these ultrasound findings was excellent.

These ultrasound findings facilitated triage and immediate decision making regarding the need for respiratory isolation in a negative pressure room without waiting for chest X-ray. Our median time to chest X-ray tripled (Table 1) during the pandemic compared to a time period prior to the pandemic. Our time to chest X-ray interpretation during the pandemic was longer than the median of 98 min reported by Zanobetti et al. in the study of emergency department lung ultrasound in non-pandemic conditions [5].

When lung consolidation with sonographic air bronchograms was visualized, point-of-care ultrasound facilitated the immediate decision to treat with antibiotics, without waiting for chest X-ray. Visualization of viral pneumonia on ultrasound may be useful to assist in the decision to initiate immediate empiric treatment with antiviral medication for future pandemic or epidemic influenza patients. In a large cohort of hospitalized H1N1 influenza A pandemic patients, only 73% of patients with radiographic evidence of pneumonia received antiviral drugs, whereas 97% received antibiotics [24]. Better recognition of viral pneumonia by ultrasound may impact outcomes, as available data have shown treatment with antiviral medication reduces mortality in hospitalized patients with influenza, even when therapy is initiated after 48 h of illness onset [24].

### Limitations

Our sample size was limited by the inability to enroll during the surge of pandemic patients due to time and resource constraints. Selection bias from convenience sampling may have occurred because patients were more likely to have been enrolled at less busier or better staffed times. In general, the patients in this series had illnesses severe enough to warrant investigation with chest X-ray. Thus, information about less ill or asymptomatic pandemic patients is lacking.

Although our calculated interobserver agreement for lung ultrasound to distinguish between viral and bacterial pneumonia is high, the number of total observations was limited, and this is reflected in our wide 95% confidence intervals. However, it is notable that our point estimate Kappa for ultrasound is higher than the reported interobserver agreement for chest X-ray for pneumonia by pediatric radiologists, 0.51 (0.39 to 0.64) [25].

Due to the large numbers of patients presenting to our emergency department during the pandemic, only hospitalized patients (four patients in our series) were confirmed with 2009 H1N1 influenza A [1]. Finding small subpleural consolidations and/or B-lines on ultrasound allows the recognition of viral pneumonia from bacterial pneumonia (lung consolidation with sonographic air bronchograms), but it is unknown if different viruses have unique lung ultrasound patterns (e.g., influenza A from RSV). We could not report test performance characteristics, such as sensitivity and specificity, as there was no practical reference gold standard for viral pneumonia at the time our study was conducted. Additionally, chest X-ray cannot be used as a gold standard for viral pneumonia. However, according to the New York City Department of Health, >90% of the circulating virus during this pandemic time period was the novel influenza A H1N1 [1].

## Conclusions

Lung ultrasound may be used to distinguish viral from bacterial pneumonia with high interobserver agreement. Lung ultrasonography may be useful during epidemics or pandemics of acute respiratory illnesses for rapid point-of-care triage and management of patients.

## Competing interests

The authors declare that they have no competing interests.

## Authors contributions

JWT and VPS participated in the design of the study, coordinated the study, and performed the statistical analysis. JWT, DOK, and VPS participated in the patient enrollment and data collection and drafting of the manuscript. All authors read and approved the final manuscript.

## Supplementary Material

Additional file 1 **Title: Small subpleural consolidation.** Description: Small subpleural consolidation consistent with viral lung ultrasound pattern. Click here for file

Additional file 2 **Title: Confluent B-lines.** Description: Confluent B-lines consistent with viral lung ultrasound pattern. Click here for file

Additional file 3 **Title: Rt anterior middle lobe lung consolidation with air bronchograms.** Description:Rt anterior middle lobe lung consolidation with air bronchograms consistent with bacterial pneumonia lung ultrasound pattern. Click here for file

Additional file 4 **Title: Confluent B-lines and lung consolidation with air bronchograms.** Description: Viral and bacterial pneumonia lung ultrasound patterns. Click here for file

## References

[B1] New York City Department of Health and Mental Hygiene Community transmission of H1N1 flu appears to decline in New York City 2009 http://www.nyc.gov/html/doh/html/pr2009/pr042-09.shtml Accessed 12 June 2010

[B2] Lessler J Reich NG Cummings DA New York City Department of Health and Mental Hygiene Swine Influenza Investigation Team Outbreak of 2009 pandemic influenza A (H1N1) at a New York City school N Engl J Med 2009 361 27 2628 2636 10.1056/NEJMoa0906089 20042754

[B3] Call SA Vollenweider MA Hornung CA Simel DL McKinney WP Does this patient have influenza? JAMA 2005 293 8 987 997 10.1001/jama.293.8.987 15728170

[B4] Lichtenstein DA Meziere GA Relevance of lung ultrasound in the diagnosis of acute respiratory failure: the BLUE protocol Chest 2008 134 1 117 125 10.1378/chest.07-2800 18403664 PMC3734893

[B5] Zanobetti M Poggioni C Pini R Can chest ultrasonography replace standard chest radiography for evaluation of acute dyspnea in the ED? Chest 2011 139 5 1140 1147 10.1378/chest.10-0435 20947649

[B6] Lichtenstein DA Lascols N Mezière G Gepner A Ultrasound diagnosis of alveolar consolidation in the critically ill Intensive Care Med 2004 30 2 276 281 10.1007/s00134-003-2075-6 14722643

[B7] Copetti R Catarossi L Ultrasound diagnosis of pneumonia in children Radiol Med 2008 113 2 190 198 10.1007/s11547-008-0247-8 18386121

[B8] Parlamento S Copetti R Di Bartolomeo S Evaluation of lung ultrasound for the diagnosis of pneumonia in the ED Am J Emerg Med 2009 27 4 379 384 10.1016/j.ajem.2008.03.009 19555605

[B9] Volpicelli G Frascisco M Sonographic detection of radio-occult interstitial lung involvement in measles pneumonia Am J Emerg Med 2009 27 1 e1 e3 128 19041553 10.1016/j.ajem.2008.03.052

[B10] Lichtenstein D Goldstein I Mourgeon E Cluzel P Grenier P Rouby JJ Comparative diagnostic performances of auscultation, chest radiography, and lung ultrasonography in acute respiratory distress syndrome Anesthesiology 2004 100 1 9 15 10.1097/00000542-200401000-00006 14695718

[B11] Volpicelli G Elbarbary M Blaivas M Lichtenstein DA Mathis G Kirkpatrick AW Melniker L Gargani L Noble VE Via G Dean A Tsung JW Soldati G Copetti R Bouhemad B Reissig A Agricola E Rouby JJ Arbelot C Liteplo A Sargsyan A Silva F Hoppmann R Breitkreutz R Seibel A Neri L Storti E Petrovic T International Liaison Committee on Lung Ultrasound (ILC-LUS) for International Consensus Conference on Lung Ultrasound (ICC-LUS) International evidence-based recommendations for point-of-care lung ultrasound Intensive Care Med 2012 38 4 577 591 10.1007/s00134-012-2513-4 22392031

[B12] Testa A Soldati G Copetti R Giannuzzi R Portale G Gentiloni-Silveri N Early recognition of the 2009 pandemic influenza A (H1N1) pneumonia by chest ultrasound Crit Care 2012 16 1 R30 10.1186/cc11201 22340202 PMC3396276

[B13] Shah VP Tunik MG Tsung JW The feasibility of diagnosing pneumonia in children with point-of-care ultrasound Pediatric Emerg Care 2009 25 10 711 712

[B14] Lichtenstein D Meziere G Biderman P Gepner A Barré O The comet-tail artifact An ultrasound sign of alveolar-interstitial syndrome. Am J Respir Crit Care Med 1997 156 5 1640 1646 10.1164/ajrccm.156.5.96-07096 9372688

[B15] Lichtenstein DA Ultrasound in the management of thoracic disease Crit Care Med 2007 35 5 Suppl S250 S261 17446785 10.1097/01.CCM.0000260674.60761.85

[B16] Weinberg B Diakoumakis EE Kass EG Seife B Zvi ZB The air bronchogram: sonographic demonstration AJR Am J Roentgenol 1986 147 3 593 595 3526846 10.2214/ajr.147.3.593

[B17] Peiris JS Yuen KY Osterhaus AD Stöhr K The severe acute respiratory distress syndrome N Engl J Med 2003 349 25 2431 2441 10.1056/NEJMra032498 14681510

[B18] Jain S Kamimoto L Bramley AM Schmitz AM Benoit SR Louie J Sugerman DE Druckenmiller JK Ritger KA Chugh R Jasuja S Deutscher M Chen S Walker JD Duchin JS Lett S Soliva S Wells EV Swerdlow D Uyeki TM Fiore AE Olsen SJ Fry AM Bridges CB Finelli L Pandemic Influenza A (H1N1) Virus Hospitalizations Investigation Team (2009) Hospitalized patients with 2009 H1N1 Influenza in the United States, April-June 2009 N Engl J Med 2009 361 1935 1944 10.1056/NEJMoa0906695 19815859

[B19] Peris A Zagli G Barbani F Tutino L Biondi S di Valvasone S Batacchi S Bonizzoli M Spina R Miniati M Pappagallo S Giovannini V Gensini GF The value of lung ultrasound monitoring in H1N1 acute respiratory distress syndrome Anaesthesia 2010 65 3 294 297 10.1111/j.1365-2044.2009.06210.x 20002364

[B20] Bramante RM Cirilli A Raio CC Point-of-care sonography in the emergency department diagnosis of acute H1N1 Influenza myocarditis J Ultrasound Med 2010 29 9 1361 1364 20733194 10.7863/jum.2010.29.9.1361

[B21] Dan D Mingsong L Jie T Xiaobo W Zhong C Yan L Xiaojin L Ming C Ultrasonographic applications after mass casualty incident cause by Wenchuan earthquake J Trauma 2010 68 6 1417 1420 10.1097/TA.0b013e3181c9b301 20234325

[B22] Sarkisian AE Khondarian RA Amirbekian NM Bagdasarian NB Khojayan RL Oganesian YT Sonographic screening of mass casualties for abdominal and renal injuries following the 1988 Armenian earthquake J Trauma 1991 31 2 247 250 1994085

[B23] Volpicelli G Noble VE Liteplo A Cardinale L Decreased sensitivity of lung ultrasound limited to the anterior chest in emergency department diagnosis of cardiogenic pulmonary edema: a retrospective analysis Crit Ultrasound J 2010 2 2 47 52 10.1007/s13089-010-0037-0

[B24] McGeer A Green KA Plevneshi A Shigayeva A Siddiqi N Raboud J Low DE Toronto Invasive Bacterial Diseases Network Antiviral therapy and outcomes of influenza requiring hospitalization in Ontario, Canada Clin Infect Dis 2007 45 12 1568 1575 10.1086/523584 18190317

[B25] Johnson J Kline JA Intraobserver and interobserver agreement of the interpretation of pediatric chest radiographs Emerg Radiol 2010 17 4 285 290 10.1007/s10140-009-0854-2 20091078

